# Cysticerci Drive Dendritic Cells to Promote *In Vitro* and *In Vivo* Tregs Differentiation

**DOI:** 10.1155/2013/981468

**Published:** 2013-05-23

**Authors:** Laura Adalid-Peralta, Asiel Arce-Sillas, Gladis Fragoso, Graciela Cárdenas, Marcos Rosetti, Didier Casanova-Hernández, Claudia Rangel-Escareño, Laura Uribe-Figueroa, Agnes Fleury, Edda Sciutto

**Affiliations:** ^1^Instituto Nacional de Neurología y Neurocirugía, Insurgentes Sur 3877, Col. La Fama, 14269 México, DF, Mexico; ^2^Unidad Periférica para el Estudio de Neuroinflamación del Instituto de Investigaciones Biomédicas de la UNAM en el Instituto Nacional de Neurología Neurocirugía, Insurgentes Sur 3877, Col. La Fama, 14269 México, DF, Mexico; ^3^Instituto de Investigaciones Biomédicas, Universidad Nacional Autónoma de México, 04510 México, DF, Mexico; ^4^Departamento de Genómica Computacional, Instituto Nacional de Medicina Genómica, INMEGEN. Periférico Sur 4809, Arenal Tepepan, 14610 México, DF, Mexico; ^5^Unidad de Genotipificación y Análisis de Expresión Affymetrix, INMEGEN, Periférico Sur 4809, Arenal Tepepan, 14610 México, DF, Mexico

## Abstract

Regulatory T cells (Tregs) play a crucial role in immune homeostasis. Treg induction is a strategy that parasites have evolved to modulate the host's inflammatory environment, facilitating their establishment and permanence. In human *Taenia solium* neurocysticercosis (NC), the concurrence of increased peripheral and central Treg levels and their capacity to inhibit T cell activation and proliferation support their role in controlling neuroinflammation. This study is aimed at identifing possible mechanisms of Treg induction in human NC. Monocyte-derived dendritic cells (DC) from healthy human donors, cocultivated with autologous CD4^+^ naïve cells either in the presence or absence of cysticerci, promoted CD25^high^Foxp3+ Treg differentiation. An increased Treg induction was observed when cysticerci were present. Moreover, an augmentation of suppressive-related molecules (SLAMF1, B7-H1, and CD205) was found in parasite-induced DC differentiation. Increased Tregs and a higher *in vivo* DC expression of the regulatory molecules SLAMF1 and CD205 in NC patients were also found. SLAMF1 gene was downregulated in NC patients with extraparenchymal cysticerci, exhibiting higher inflammation levels than patients with parenchymal parasites. Our findings suggest that cysticerci may modulate DC to favor a suppressive environment, which may help parasite establishment, minimizing the excessive inflammation, which may lead to tissue damage.

## 1. Introduction

Natural (thymic) and inducible regulatory T cells (Tregs) play a pivotal role in maintaining the immune system homeostasis. While natural Tregs are produced in the thymus at any time, inducible Tregs acquire a regulatory function in the context of a given infection or a neoplastic process [1]. A variety of inducible Treg subpopulations mediating their immune suppressive effects by different mechanisms have been reported [2–4]. Disregarding their effectiveness in controlling inflammation, it is conceivable that Tregs could promote a more permissive environment for parasite establishment [5, 6]. The increased Treg levels found in many different protozoa and cestode infections are consistent with this possibility [7, 8].

The role of Tregs in neurocysticercosis (NC), a parasitic disease caused by the establishment of *Taenia solium* metacestode in the central nervous system (CNS), begins to be explored. Increased central and peripheral Treg levels were observed in severe NC patients. These Tregs seem to participate in the control of the inflammatory response, since a negative correlation between the percentage of peripheral Tregs and activated CD8+ and CD4^+^ T cells, along with a depressed T cell proliferative response, was observed [9]. However, the mechanisms underlying Treg induction in NC are still unknown.

Dendritic cells play a prominent role in Treg induction in the beginning of the immune response to pathogens, either by promoting the conversion of naïve T cells to Treg subpopulations or by expanding the population of preexisting Treg cells [10]. Mature DCs direct conventional CD4^+^ cells to become either specific T helper or T regulatory cell subsets, depending on the affinity of their TCR to the antigen, the strength of the costimulatory signals provided by antigen-presenting cells (APCs), and the cytokine milieu. In addition, the absence of inflammation arrests dendritic cells into an immature or semimature state (iDC), which promotes T cell tolerance by conversion of naïve T cells into Tregs [11]. iDCs are characterized by expressing MHC II, by a high phagocytosis capacity, and by low CD80/CD86 expression. iDCs also produce IL-10, but neither IL-12 nor TNF*α* [10, 12]. Moreover, it has been proposed that preexisting Tregs can educate iDCs to become tolerogenic, promoting Treg generation [10, 13]. Helminths, as well as other pathogens, may modulate the immune tolerance properties of DCs [14–17].

This study was designed to evaluate the capacity of cysticerci to modulate dendritic cells, driving them to Treg induction, both *in vitro* and *in vivo*.

## 2. Materials and Methods

### 2.1. Parasites

*Taenia solium* metacestodes were obtained from naturally infected pigs coming from villages of Guerrero, an endemic region in Mexico. Pigs were euthanized according to ethical animal handling regulations in Mexico. Cysticerci were individually harvested from muscle tissue and maintained in RPMI 1640 medium containing 10% fetal calf serum and 1% antibiotics (Invitrogen, NY, USA). Cysticerci of *Taenia crassiceps ORF* strain were also used in this study to evaluate their potential to induce iDCs, and therefore Tregs. *T. crassiceps* cysticerci were obtained from infected BALB/cAnN female mice. Parasites were harvested from the peritoneal cavity of stock female mice after 10 weeks of infection.

### 2.2. Cell Purification and DC Generation in the Presence of Live Cysticerci

Human cells were isolated from buffy coats from blood of healthy donors (Banco de Sangre del Centro Médico Nacional Siglo XXI, México, DF).

A RosetteSep Human CD4^+^ T Cell Enrichment Cocktail kit was used for CD4^+^ T cell purification (StemCell, Vancouver, Canada). Purified CD4^+^ T lymphocytes including >90% of CD3+ CD4^+^ cells were kept frozen at –80°C until used.

Monocyte-derived DCs (MDDCs) were isolated from mononuclear cells from healthy donors using a RosetteSep Human Monocyte Enrichment Cocktail kit (StemCell, Vancouver, Canada). MDDCs were generated as previously reported [18] with only minor modifications. Briefly, monocytes were plated in Petri dishes (P100) at 5 × 10^5^ cells/mL in 10 mL of RPMI 1640 medium (Invitrogen, NY, USA) containing 10% human AB serum (HSAB) and 1% antibiotics, 20 ng/mL IL-4 (eBiosciences CA, USA), and 100 ng/mL granulocyte-macrophage colony-stimulating factor (eBiosciences, San Diego, CA, USA) for 8 days. One half of complete medium, including cytokines, was replaced at day 4.

To evaluate the role of cysticerci during DC differentiation, monocytes were cultured as described above, either with or without vesicular *Taenia crassiceps* (40 cysts) or *Taenia solium* (5 cysts) cysticerci per dish. The effect of both cestodes on DC differentiation was tested considering that murine cysticerci may eventually provide a controlled parasite source of purified immune-modulatory components. Dexamethasone at 10^-7 ^M was used as a positive control.

### 2.3. *In Vitro* Treg Induction with Cysticercal Antigens

Parasite-induced MDDCs were seeded at 2.5 × 10^4^ cell/mL in 1 mL RPMI 1640 medium (Invitrogen, NY, USA) containing 10% human AB serum and 1% antibiotics (Invitrogen, NY, USA). MDDCs were maintained in culture for 24 h. During this period, dexamethasone at 10^-7 ^M, which maintains a tolerogenic DC phenotype, was used as control [19]. Parasite-induced DCs were pulsed using 3 *μ*g/mL of total antigens from *T. crassiceps* cysticerci. *T. crassiceps* antigens were employed considering that *T. solium* and *T. crassiceps* cysticerci share in common more than 98% of the expressed antigens [20]. 1.25 × 10^5^ autologous CD4^+^ T lymphocytes were cocultured for 7 days with MDDCs in RPMI 1640 medium (Invitrogen, NY, USA) containing 10% human AB serum and 1% antibiotics (Invitrogen, NY, USA). Then, the phenotype of regulatory T cells was evaluated by flow cytometry as described below. Supernatants of these cultures were used to evaluate cytokine profile.

### 2.4. Cell Phenotype

The following combination of antibodies was used to characterize the phenotype of MDDCs: (a) MHC-II FITC (mouse IgG2a k), SLAMF1 PE (mouse IgG1 k), CD11c PerCP-eFluor 710 (mouse IgG1 k), and B7/H1 APC (mouse IgG1 k); (b) CD83 FITC, CD80 PE, CD11c PerCP-eFluor 710 (mouse IgG1 k), and CD86 APC (mouse IgG1 k); (c) CD205 FITC (mouse IgG2b k), CD40 PE (mouse IgG1 k), CD11c PerCP-eFluor 710 (mouse IgG1 k), and ILT3 APC (mouse IgG1 k). Inducible regulatory T cell phenotype was assessed using CD127 FITC (mouse IgG1 k), Foxp3 PE (Rat IgG2a k), CD4 PerCp (mouse IgG1 k), and CD25 APC (mouse IgG1 k). Regulatory T cells were intracellularly stained for Foxp3 using the eBiosciences kit (most antibodies used for cell staining were purchased from eBiosciences, CA, USA). All antibodies were titrated for optimal detection of positive populations prior to its use, considering the manufacturer's recommended concentrations.

For cytometry analyses, cells were first gated according to lymphocyte forward and side light-scattering properties. Treg numbers were defined as the fraction of Foxp3+ cells among CD4^+^ CD25^high^ cells.

### 2.5. Cytokines

Cytokine levels in supernatants were measured using the cytometric bead array Cytokine Kit II (BD Biosciences Pharmingen, CA, USA), according to the manufacturer's instructions, in an FACSCalibur cytometer. Data were analyzed with the BD Cytometric Bead Array software (BD Biosciences Pharmingen, CA, USA). Flow cytometer was calibrated using BD FACSComp (BD Biosciences Pharmingen, CA, USA) and BD CaliBRITE beads (BD Biosciences Pharmingen, CA, USA). Assay sensitivities were set as follows: IL-2 (2.6 pg/mL), IL-6 (3.0 pg/mL), IL-10 (2.8 pg/mL), TNF-*α* (2.8 pg/mL), and IFN-*γ* (7.1 pg/mL). TGF-*β* were measured using the human/mouse TGF-beta 1 ELISA Ready-SET-Go (eBiosciences, CA, USA). ELISA was performed according to the manufacturer's instructions; detection limit was 60 pg/mL. All samples were run in duplicate.

### 2.6. DC and Treg Phenotype in NC Patients

Blood samples from 13 patients who attended at the Instituto Nacional de Neurología y Neurocirugía and Centro Médico Nacional Siglo XXI in Mexico City with confirmed NC diagnosis were included in this study. All samples were taken before any treatment was administered. Eight male (age mean 56.25 ± 3.8 years) and five female (age mean 37 ± 1.6 years) patients were included in this study.

In all cases, NC was diagnosed based on clinical manifestations (seizures, focal deficit, and intracranial hypertension) and radiological studies such as MRI and computed tomography (CT).

Vesicular parasites were observed in most cases (12/13 patients). A solitary cysticercus was seen in 9 patients, and multiple cysticerci in 2 patients; the rest of the patients had calcified and colloidal forms. In seven patients, parasite was located at the subarachnoid space of the base (SAB), while the rest of the patients showed parasites at the cerebral parenchyma (P). Patients with SAB cysticerci exhibited inflammatory traits in cerebral spinal fluid (glucose 30.7 ± 28 mg/dL, proteins 421 ± 407 mg/dL, and 70.7 ± 96.9 cell/mm^3^). In contrast, patients with parenchymal cysticerci showed no inflammation signs. Blood samples from 5 healthy subjects were included as controls.

### 2.7. Microarrays

DNA microarrays were used to compare the gene expression profile of mononuclear peripheral cells (PBMCs) from a cohort of 8 NC patients, 40 ± 14 years old. PBMC from all patients were obtained 8 days after cysticidal treatment with albendazole, 30 mg per kg, and concomitant corticosteroid administration. These 8 (4 females and 4 males) patients showed cysticerci in different locations. Cysticerci were established in the subarachnoid basal space or in the ventricles (SAB/IV) in 5 patients and were established in the cerebral parenchyma (P) in 3 patients. When required for patients' followup, CSF was obtained by lumbar puncture at the National Institute of Neurology and Neurosurgery of Mexico City. A mean of 74 cell/mm^3^ (range 1–261) was found in SAB/IV-NC patients, and a mean of 6.6 cell/mm^3^ (range 0–18) cells was found in P-NC patients. PBMCs were isolated and then cultured by 72 h with *T. solium* cysticercal antigens, obtained as previously described [21].

Recovered cells were placed in TRIzol (Gibco, NY, USA) for RNA purification using the RNeasy mini kit (QIAGEN, TX, USA) following the manufacturer's instructions. The amount of obtained RNA was estimated in an Agilent 2100 Bioanalyzer (Agilent Technologies, Waldbronn, Deutschland). Comparisons were done with respect to parasite location (SAB/IV-NC versus P-NC) using the R-language platform, under the terms of the Free Software Foundations GNU General License (see http://cran.r-project.org/). Data were standardized and normalized. All genes were analyzed using the Reactome software (see http://www.reactome.org/) to find a possible biological pathway involved.

### 2.8. Ethical Considerations

The present study fulfilled all regulations for research with human subjects as required by the Mexican law and international regulations. It also complied with all ethical aspects considered in the General Rules of Health for Clinical Investigation. Ethics Committee at Instituto Nacional de Neurología y Neurocirugía, México approved the protocol. Written informed consent was obtained from all participants. Patients were informed that samples obtained would be used for this work.

### 2.9. Statistical Analysis

Data were processed in InStat (GraphPad Software Inc., CA, USA). Variables were described using mean ± SD. Differences between groups were calculated with Students' *t*-test. *P* values less than 0.05 were considered significant.

## 3. Results

### 3.1. Effect of Cysticerci on Dendritic Cell Phenotype

**Figure 1:**
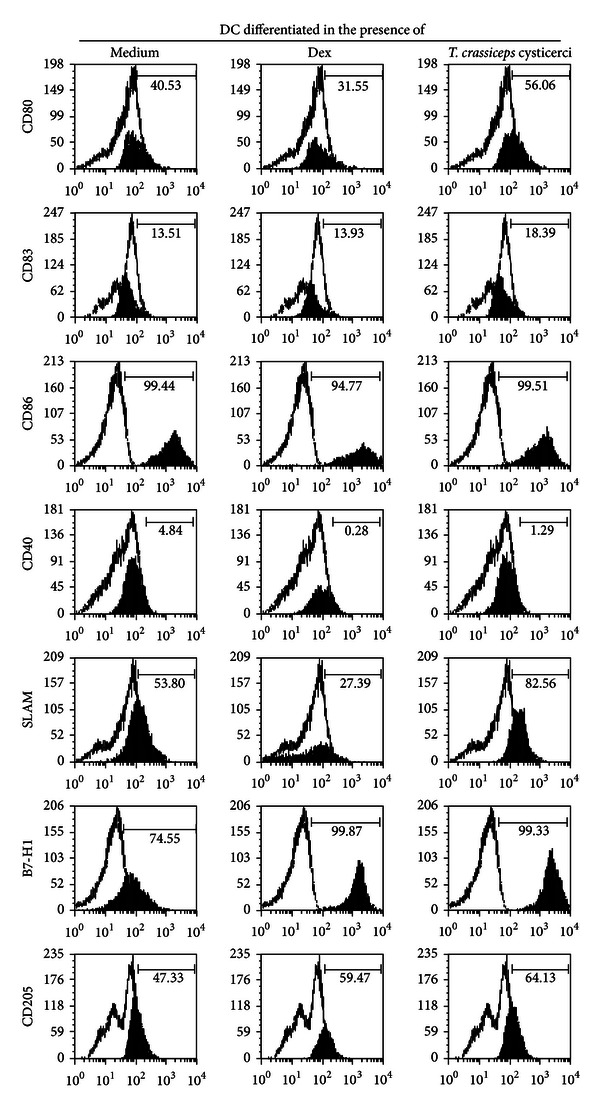
Parasite effect on DC differentiation and activation. Monocytes were differentiated to DC in presence of IL-4 and GM-CSF, plus medium or dexamethasone or *Taenia crassiceps* cysticerci. On day 8, the DC phenotype was studied. Representative data of three independent experiments are shown. Expression of CD80, CD83, CD86, CD40, SLAMF1, B7-H1, and CD205 gated on CD11c+ cells was analyzed by FACS. The isotype controls are shown in white histograms.

The semimature DC phenotype is associated with tolerance and Treg induction [12]. To evaluate the effect of cysticerci on dendritic cell differentiation, the phenotype of DC generated from CD14+ monocytes either in the presence or absence of vesicular cysticerci after 8-day culture was studied. Dexamethasone (10^-7 ^M) was employed as a positive control (Figure 1). The expression of CD83+, as well as the costimulatory (CD80, CD86, CD40, HLA-DR) and regulatory molecules (SLAMF1, B7-H1, CD205, and ILT3) on CD11c+, was measured. As shown in Figure 1, a higher expression of SLAMF1, B7-H1, and CD205 was observed in cells differentiated in the presence of cysticerci. The expression of HLA-DR, CD80, CD83, CD86, CD40, and ILT3 molecules did not differ between control and parasite-driven differentiated DC cells. On the other hand, CD80, CD40, and SLAMF1 were diminished in dexamethasone-treated cells with respect to control (Figure 1). HLA-DR and ILT3 expression did not differ from control at any tested condition (data not shown). Altogether, these results indicate that parasite promotes the expression of molecules related to a DC tolerogenic phenotype. 

### 3.2. *Taenia Solium* and *T. crassiceps* Cysticerci Promote *In Vitro* Regulatory T Cells Differentiation

**Figure 2:**
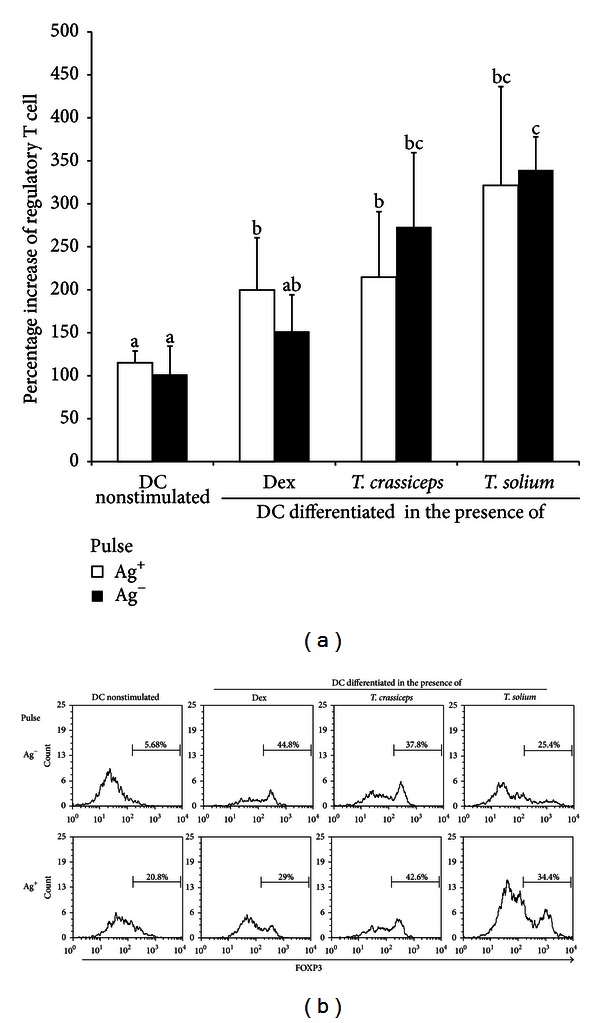
*In vitro* differentiation of dendritic cells in the presence of *Taenia solium* or *T. crassiceps* cysticerci promotes Treg induction. (a) Increased percentage in Treg cell induction. Percentage was calculated as follows: (percentage of Tregs induced/percentage of basal Tregs) × 100. Different letters indicate significant differences between groups at *P* < 0.05. (b) Representative histograms showing Foxp3 induction within CD4^+^ CD25^high^ cells. Data are representative of three independent experiments.

We investigated whether differentiation *in vitro* in the presence of live cysticerci stimulated the differentiation to CD4^+^ CD25^high^ Foxp3+ Tregs. Human DCs, either differentiated in the presence or absence of cysticerci, were pulsed or not with cysticercal proteins and used to stimulate autologous CD4^+^ T lymphocytes. Seven days later, the expression of CD25^high^ and Foxp3 within CD4^+^ T lymphocytes was measured. As a positive control of Treg induction, differentiated DCs were treated with dexamethasone (10^-7 ^M). As shown in Figure 2, cocultivation with cysticerci significantly increased the percentage of CD4^+^ lymphocytes coexpressing the CD25^high^ and Foxp3 fraction with respect to non-stimulated DC, from 115 ± 13.7 to 214.6 ± 76.3 when cocultured with *T. crassiceps* (*P* = 0.04) and to 321.2 ± 115 (*P* = 0.05) when cocultured with *T. solium* cysticerci. When DCs were pulsed with parasite antigens, Treg cells were also significantly increased, from 101.2 ± 33 to 273 ± 86.5 (*P* = 0.01) with *T. crassiceps-*differentiated DC cells, and to 339.3 ± 38.6 (*P* = 0.0002) with *T. solium-*differentiated DC cells (Figure 2).

### 3.3. Cytokine Profile

The levels of induced cytokines were measured in supernatants from *in vitro* DC differentiation after 8 days of culture and from *in vitro* Treg induction after 7 days of culture. During DC differentiation, only IL-10 was significantly increased when cells were differentiated in presence of dexamethasone (Table 1). No difference was observed in the other tested conditions.

### 3.4. Dendritic Cells and Regulatory T Cells in NC Patients

The *in vivo* effect of parasite products on the phenotype of peripheral DC from NC patients was studied. The percentage of DC and the expression of regulatory (SLAMF1, CD205, and ILT3) and costimulatory molecules (HLA-DR, CD86, and CD40) in CD11c cells in 13 NC patients and 5 healthy subjects was measured. As Table 2 shows, SLAMF1, and CD205 are significantly increased in DC from NC patients from 3.8 to 9.7 and from 4.3 to 21.5, respectively (*P* < 0.05). Moreover, peripheral Tregs are also increased in NC patients from 4.35 to 14.22.

### 3.5. SLAMF1 Is Downregulated in PBMC from Patients with SAB/IV Parasites

As Table 3 shows, only four immune-related genes of the 32,322 included in the array were found downregulated in severe SAB/IV-NC patients with respect to P-NC patients. Among them figures the SLAMF1 gene, whose expressed protein was also found in dendritic cells from non-treated NC patients (Table 2). The other downregulated genes found in these patients were MTOR, NFKB2, and IL12RB2 (Table 3). In these severe SAB/IV-NC patients, TGB2 and IL24 genes were found to be up-regulated.

## 4. Discussion

Tregs play a pivotal role in modulating the host environment, so parasites may find more appropriate conditions for their establishment and development [7]. It is also possible that Tregs may favor the parasite persistence even when a specific treatment is used to promote the destruction of the parasite [22]. During *Taenia solium* infection, an increase in the levels of regulatory T cells in blood and CSF in NC patients has been observed, probably promoted by the parasite for controlling the central inflammatory environment and thus favoring its survival [9]. Increased Tregs in NC patients may result from expanded natural Tregs or may be induced by the parasite. The latter possibility was explored herein. On the other hand, DCs may drive T cell differentiation to regulatory or effector cells, depending on their activation status. In this study, *in vivo* and *in vitro* lines of evidence point to the effect of cysticercal components on the activation of dendritic cells and their impact on promoting T cell differentiation to CD25^high^ Foxp3+ CD4 T cells. Indeed, co-cultivation of dendritic cells with either *T. solium* or *T. crassiceps* cysticerci promotes a status that favors the differentiation of peripheral T cells to CD4^+^ Tregs. Comparable results were found when peripheral cells from non-treated NC patients were studied.

The similar effects induced by the presence both of *T. solium* or *T. crassiceps* cysticerci merit some comments. It is well known that both cestodes share multiple antigens [23], a fact that has been exploited to use the murine cysticercal antigens for diagnosis [24, 25]. 

Parasite-differentiated dendritic cells show no difference with respect to control in the maturation marker (CD83), neither in the costimulatory molecules HLA-DR, CD80, CD86, or CD40; these findings are compatible with an immature dendritic phenotype. In contrast, an overexpression of SLAMF1, B7-H1, and CD205 was observed. The latter two molecules are related to a tolerogenic DC phenotype, as reported in many studies [12, 26]. It is important to note that the expression of SLAMF1, B7-H1, and CD205 was found accompanied by Treg induction in this study, as well. However, their participation in Treg induction remains to be elucidated. This may be particularly relevant since no increase in the two main regulatory cytokines, IL10 and TGF*β*, was observed in the supernatants recovered during *in vitro* Treg induction (Table 1). An increased expression of SLAMF1 and CD205 in dendritic cells and in CD25^high^ Foxp3+ CD4 T cells was observed in NC patients. Both *in vitro* and *in vivo* findings reinforce the relevance of SLAMF1 and CD205 dendritic cells, defining their critical role in T cell immunity regulation [27, 28].

It is also worth noticing the downexpression of SLAMF1 in the group of 8 SAB/IV-NC patients with respect to those patients harboring cysticerci in the parenchyma. This apparent discordance with the *in vitro* results from nontreated NC patients can be traced to differences promoted by *in vivo* or *in vitro* conditions, which may differentially modulate the expression of the SLAM-associated protein (SAP) in lymphocytes. Indeed, the expression of this adaptor SAP protein promotes the inflammatory response, while its absence promotes a regulatory environment [28]. SLAM-family receptors presenting cells ligands carry out important immunomodulatory functions: by one side, they regulate lymphocyte interactions and adhesion [28, 29] as well as a tolerogenic immune profile [11, 12]. Although there is no previous information about the functions of SLAM receptors during NC, the results shown in this study point to their possible participation in modulating the inflammatory response promoted by cysticidal and/or corticosteroid treatments. Changes found in MTOR, IL12RB2, NFKB2, and TGF-*β* genes match with an immunoregulatory environment. MTOR [30], IL12RB2 [31], and NFKB2 [32, 33] genes, coding for proteins that promote an inflammatory environment, are found downregulated, whilst TGF-*β*, which promotes a regulatory immune response [7], was up-regulated.

## 5. Conclusions

Overall, the results shown in this study reinforce our previous findings on the relevance of Tregs in controlling the extent of the inflammatory response in NC patients, and added *in vivo* and *in vitro* lines of evidence that cysticerci may drive a particular dendritic cell phenotype that induces regulatory T cells, even though the mechanisms that underlined this phenomenon remain to be elucidated. Additionally, while the clinical relevance of the promotion of this regulatory environment needs to be evaluated, it is feasible to propose that it could favor parasite survival.

## Figures and Tables

**Table 1:** Levels of cytokines in supernatants from *in vitro* DC differentiation and *in vitro* regulatory T cell induction.

	IFN-*γ*	TNF-*α*	IL-10	IL-6	IL-2	TGF-*β*
DC nonstimulated	0.39 ± 0.88	3.98 ± 3.82	3.17 ± 1.79	>5000	0.34 ± 0.77	406.50 ± 191.69
^†^Control medium 10% HSAB	3.55 ± 0.51	5.78 ± 1.79	5.12 ± 1.50	>5000	1.59 ± 1.41	ND
^b^DC differentiation in the presence of						
Dexamethasone	0	3.12 ± 0.47	30.84 ± 5.82^a^	>5000	0	545.17 ± 289.37
*Taenia crassiceps *	0.78 ± 1.75	2.98 ± 1.75	3.77 ± 1.76	>5000	0.72 ± 0.98	341.50 ± 238.19
*Taenia solium *	0	4.58 ± 4.44	7.31 ± 4.88	>5000	0	436.00 ± 243.95
Treg induction from naïve CD4^+^ cells						
DC nonstimulated, nonpulsed (Ag^−^)	0.76 ± 1.31	1.21 ± 1.07	0.99 ± 0.93	2.58 ± 0.70	0.45 ± 0.78	124.60 ± 5.35
DC nonstimulated pulsed (Ag^+^)	0.78 ± 1.75	2.06 ± 0.88	1.22 ± 1.12	2.78 ± 0.60	0.43 ± 0.95	93.07 ± 16.98
Dexamethasone nonpulsed (Ag^−^)	0.63 ± 1.26	1.68 ± 0.34	1.01 ± 1.18	2.97 ± 2.02	0.39 ± 0.78	95.49 ± 19.66
Dexamethasone pulsed (Ag^+^)	1.00 ± 1.74	2.49 ± 0.36	2.32 ± 1.36	2.78 ± 1.10	1.38 ± 1.27	98.63 ± 26.39
*T. crassiceps* nonpulsed (Ag^−^)	1.23 ± 2.13	2.09 ± 0.79	1.02 ± 1.76	8.15 ± 1.26	1.31 ± 1.21	99.22 ± 31.64
*T. crassiceps* pulsed (Ag^+^)	1.26 ± 1.81	2.18 ± 0.38	1.44 ± 0.81	29.34 ± 46.75	0.43 ± 0.95	119.80 ± 42.56
*T. solium* nonpulsed (Ag^−^)	4.50 ± 2.61	2.35 ± 0.68	1.48 ± 2.10	56.99 ± 5.21	12.14 ± 11.79	111.00 ± 3.54
*T. solium* pulsed (Ag^+^)	1.73 ± 2.45	2.28 ± 0.68	1.95 ± 0.09	11.68 ± 6.02	6.61 ± 6.16	78.50 ± 21.21
Control T cell	1.54 ± 1.41	1.90 ± 0.48	1.77 ± 0.99	2.01 ± 0.45	0.88 ± 1.20	101.00 ± 16.58

^a^Significantly different (*P* < 0.05) compared to nonstimulated DC. ^b^Human  AB sera from healthy donors were used in DC differentiation experiments. ^†^Levels of cytokines in the control medium supplemented with 10% HSAB are shown.

**Table 2:** Phenotype of peripheral dendritic and regulatory T cells in NC patients and healthy subjects.

Phenotype	NC patients	Healthy subjects	*P *
Dendritic cells			
SLAM^+^CD11C^+^	9.65 ± 5.66	3.80 ± 1.88	0.045
CD205^+^CD11C^+^	21.55 ± 13.64	4.27 ± 3.12	0.014
ILT3^+^CD11C^+^	35.01 ± 27.61	31.48 ± 28.70	0.813
HLA-DR^+^CD11C^+^	71.47 ± 19.87	69.05 ± 12.96	0.805
CD86^+^CD11C^+^	39.70 ± 25.87	54.21 ±12.45	0.250
CD40^+^CD11C^+^	7.92 ± 7.98	2.81 ± 1.49	0.182
T cells			
CD4^+^CD25^ High^ FoxP3^+^CD127^−/low^	14.22 ± 9.08	4.35 ± 4.26	0.0194
CD4^+^T lymphocytes	30.85 ± 11.49	43.59 ± 5.60	0.07

**Table 3:** Genes differentially expressed in NC patients with respect to the parasite location after 8 days of treatment.

	NC caused by cysticerci localized in the subarachnoid base or intraventricular versus parenchymal		
	Genes	Log	*B*
Downregulated	MTOR	−0.69	0.95
	NFKB2	−0.93	0.78
	SLAMF1	−1.05	2.18
	IL12RB2	−0.81	1.19
Upregulated	IL24	0.82	0.96
	TGFB2	1.06	2.13

The log fold change (Log FC) describes how much a quantity changes from an initial to a final value in a determinate gene. *B* value represents the possibility that the gene is differentially expressed.
